# Efficacy of a Cell-Cycle Decoying Killer Adenovirus on 3-D Gelfoam^®^-Histoculture and Tumor-Sphere Models of Chemo-Resistant Stomach Carcinomatosis Visualized by FUCCI Imaging

**DOI:** 10.1371/journal.pone.0162991

**Published:** 2016-09-27

**Authors:** Shuya Yano, Kiyoto Takehara, Hiroshi Tazawa, Hiroyuki Kishimoto, Yasuo Urata, Shunsuke Kagawa, Toshiyoshi Fujiwara, Robert M. Hoffman

**Affiliations:** 1 AntiCancer, Inc., San Diego, California, United States of America; 2 Department of Surgery, University of California San Diego, San Diego, California, United States of America; 3 Department of Gastroenterological Surgery, Okayama University, Graduate School of Medicine, Dentistry and Pharmaceutical Sciences, Okayama, Japan; 4 Center for Innovative Clinical Medicine, Okayama University Hospital, Okayama, Japan; 5 Oncolys BioPharm Inc., Tokyo, Japan; Virginia Commonwealth University, UNITED STATES

## Abstract

Stomach cancer carcinomatosis peritonitis (SCCP) is a recalcitrant disease. The goal of the present study was to establish an in vitro-in vivo-like imageable model of SCCP to develop cell-cycle-based therapeutics of SCCP. We established 3-D Gelfoam^®^ histoculture and tumor-sphere models of SCCP. FUCCI-expressing MKN-45 stomach cancer cells were transferred to express the fluorescence ubiquinized cell-cycle indicator (FUCCI). FUCCI-expressing MKN-45 cells formed spheres on agarose or on Gelfoam^®^ grew into tumor-like structures with G_0_/G_1_ cancer cells in the center and S/G_2_ cancer cells located in the surface as indicated by FUCCI imaging when the cells fluoresced red or green, respectively. We treated FUCCI-expressing cancer cells forming SCCP tumors in Gelfoam^®^ histoculture with OBP-301, cisplatinum (CDDP), or paclitaxel. CDDP or paclitaxel killed only cycling cancer cells and were ineffective against G_1_/G_2_ MKN-45 cells in tumors growing on Gelfoam^®^. In contrast, the telomerase-dependent adenovirus OBP-301 decoyed the MKN-45 cells in tumors on Gelfoam^®^ to cycle from G_0_/G_1_ phase to S/G_2_ phase and reduced their viability. CDDP- or paclitaxel-treated MKN-45 tumors remained quiescent and did not change in size. In contrast, OB-301 reduced the size of the MKN-45 tumors on Gelfoam^®^. We examined the cell cycle-related proteins using Western blotting. CDDP increased the expression of p53 and p21 indicating cell cycle arrest. In contrast, OBP-301 decreased the expression of p53 and p21 Furthermore, OBP-301 increased the expression of E2F and pAkt as further indication of cell cycle decoy. This 3-D Gelfoam^®^ histoculture and FUCCI imaging are powerful tools to discover effective therapy of SCCP such as OBP-301.

## Introduction

Collagen sponge-gel histoculture was developed by Leighton [1] and enables cultured cells to form 3-dimensional structures [2].

As described in detail previously [2], osteosarcoma growing on Gelfoam^®^, formed three-dimensional tissue-like structures, but did not form structures in monolayer culture and only colonies on Matrigel^TM^ [2,3].

As also previously described in detail [3–11], Gelfoam^®^ can be used for tumor growth [4, 5], to culture differentiating stem cells [6–8], to culture hair-producing hair follicles [9], to culture hair-growing skin [10] and to culture functional lymphoid tissue [11].

We recently observed that cancer cells in G_0_/G_1_ phase in Gelfoam^®^ histoculture had more extensive migration than in S/G_2_/M. Upon entry into S/G_2_/M, the cells ceased migrating. After mitosis, when the cells entered G_0_/G_1_, the cells could resume migrating. The migrating cells in G_0_/G_1_ were not sensitive to chemotherapy. Fluorescence ubiquination cell cycle indicator (FUCCI) enabled monitoring of the cell cycle phase of each cell in real time as described in detail previously [12, 13].

We previously showed with FUCCI imaging that cancer cells in Gelfoam^®^ and in tumors in mice had a similar cell-cycle-phase distribution. Only the surface cells in Gelfoam^®^ histoculture and tumors was cycling; interior cells of tumors and Gelfoam^®^ histocultures were quiescent in G_0_/G_1_. In mono-layer culture, in contrast, cancer cells continuously cycle. In both Gelfoam^®^ histoculture and tumors in mice, chemotherapy had similar efficacy, in contrast to cancer cells in mono-layer, which were much more chemo-sensitive as described previously in detail [13–15].

In the present report, we determined the efficacy of cell-cycle decoying telomerase-dependent adenovirus OBP-301 on stomach cancer carcinoma growing on Gelfoam^®^ histoculture, using FUCCI imaging to monitor cell-cycle changes.

## Materials and Methods

### Cells

MKN-45 cells (Cat. #JCRB 0254; Japanese Collection of Research Bioresources, Osaka, Japan), expressing FUCCI, were cultured on plastic plates in RPMI-1640 medium to produce sufficient numbers of cells for Gelfoam^®^ histoculture (please see below) [12, 14, 16, 17].

### Gelfoam^®^ Histoculture

Gelfoam^®^ (Pharmacia & Upjohn, Kalamazoo, MI), was cut into 1 cm cubes and placed in 6-well tissue culture plates containing RPMI-1640 medium and placed in a 37°C incubator until the Gelfoam^®^ absorbed the medium. FUCCI-expressing cancer cells (1×10^6^) were then carefully layered on top of the Gelfoam^®^ and placed in an incubator for 1 h, after which additional medium was added up to the top of the Gelfoam^®^ and then incubated again at 37°C with 5% CO_2_/95% air, as described previously in detail [2, 4–6, 12, 18].

### Establishment of FUCCI-Expressing MKN-45 Cells

In order to establish FUCCI-expressing MKN-45 cells, two plasmids were utilized: mKO2-hCdt1 containing an orange-red fluorescent protein and mAG-hGem, containint a green fluorescent protein (Medical and Biological Laboratory, Nagoya, Japan) [19] were sequentially tranfected into MKN-45 cells with the use of Lipofectamine™ LTX (Invitrogen, Carlsbad, CA). After transfection with each plasmid, the cells were cultured for appropriate periods of time and sorted for the fluorescence color corresponding to plasmid used as described previously in detail [12].

### Imaging of FUCCI-Expressing MKN-45 Cells

The OV100 variable magnification fluorescence imaging system (Olympus) was used to acquire macro images as described previously in detail [20]. The FV1000 confocal laser scanning microscope (Olympus) was used to image single FUCCI-expressing cells. The FV1000 contains 473 nm and 559 nm lasers [21]. The 4× objective lens with a 0.20 numerical aperature and the 20× objective lens with a 0.95 numerical aperature were used. Fluoview software (Olympus) was used for image acquisition and Velocity 6.0 version (Perkin Elmer) [15] were used for 3D analysis. The above methods were previously described in detail [21].

### Statistical Analysis

The Student’s *t*-test was used for comparison of 2 groups. Data are presented as means ± SD. Significance was determined via a *P* value of < 0.05 [12].

## Results and Discussion

### Cell-cycle Decoy and Killer Efficacy of Telomerase-Dependent Adenovirus OBP-301 in Comparison with Chemotherapy on Tumor Spheres

**Fig 1:**
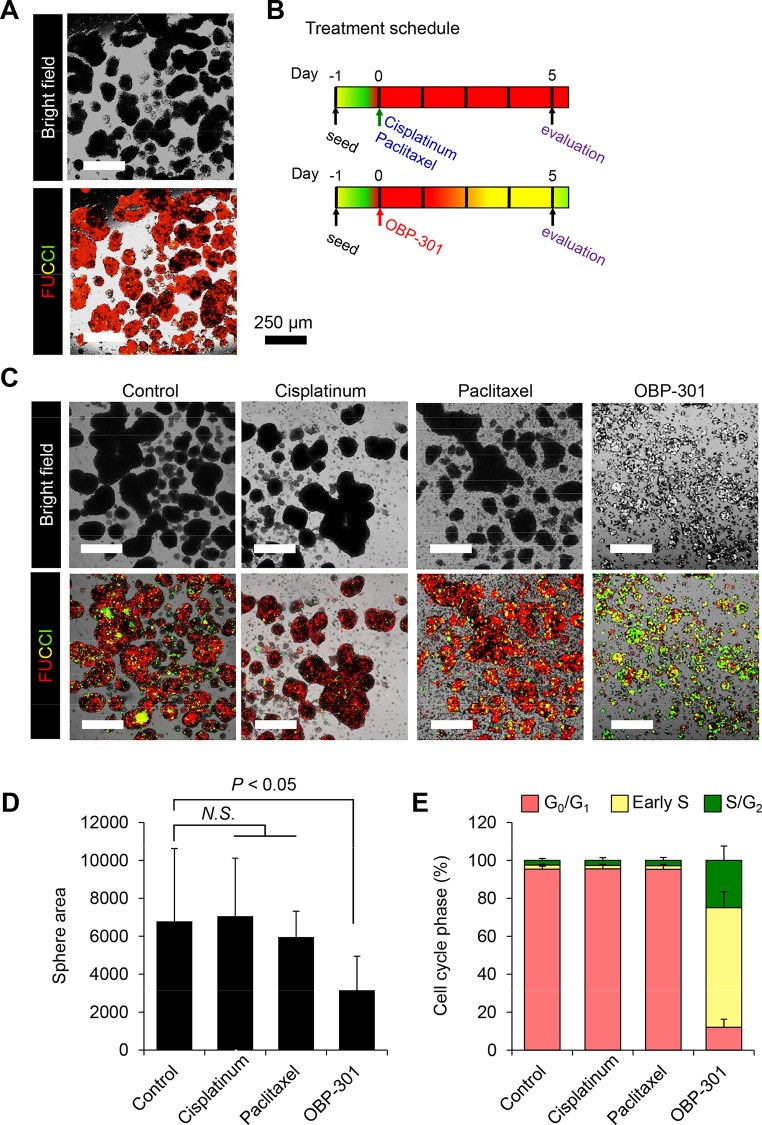
OBP-301 decoys MKN-45 stomach cancer cells in G_1_/G_0_ to cycle in tumor spheres. **A.** Representative of FUCCI MKN-45 stomach cancer cells *in vitro*. **B.** Experimental setup for treatment of FUCCI-expressing MKN-45 cells in spheres with OBP-301, cisplatinum (CDDP), or paclitaxel. **C.** Representative images of control, OBP-301-, CDDP- or paclitaxel-treated spheres. **D.** Bar graphs show the size of viable tumor spheres after each treatment. **E.** Histogram shows cell cycle phase of treated tumor spheres. Data are shown as means ± SD (n = 5). *P < 0.01.

FUCCI-expressing MKN-45 stomach cancer cells cultured on agarose spontaneously formed spheres and were mostly FUCCI red, indicating they were in G_0_/G_1_ phase (Fig 1A). Tumor spheres were treated with OBP-301, cisplatinum (CDDP), or, paclitaxel (Fig 1B). CDDP- or paclitaxel-treated MKN-45 cells remained in the quiescent G_0_/G_1_ state and did not change size compared with control-spheres or spheres before treatment (Fig 1C–1E). In contrast, OBP-301 decoyed the cell cycle of tumor spheres from G_0_/G_1_ phase to S/G_2_ phase where they expressed FUCCI green and reduced the size of the tumor (Fig 1C–1E). G_0_/G_1_ cancer cells in spheres were resistant to chemotherapy but could be decoyed to S/G_2_ by OBP-301 which reduced their viability.

### Cell-cycle Distribution of MKN-45 Stomach Cancer Cells Forming Tumors on Gelfoam^®^

**Fig 2:**
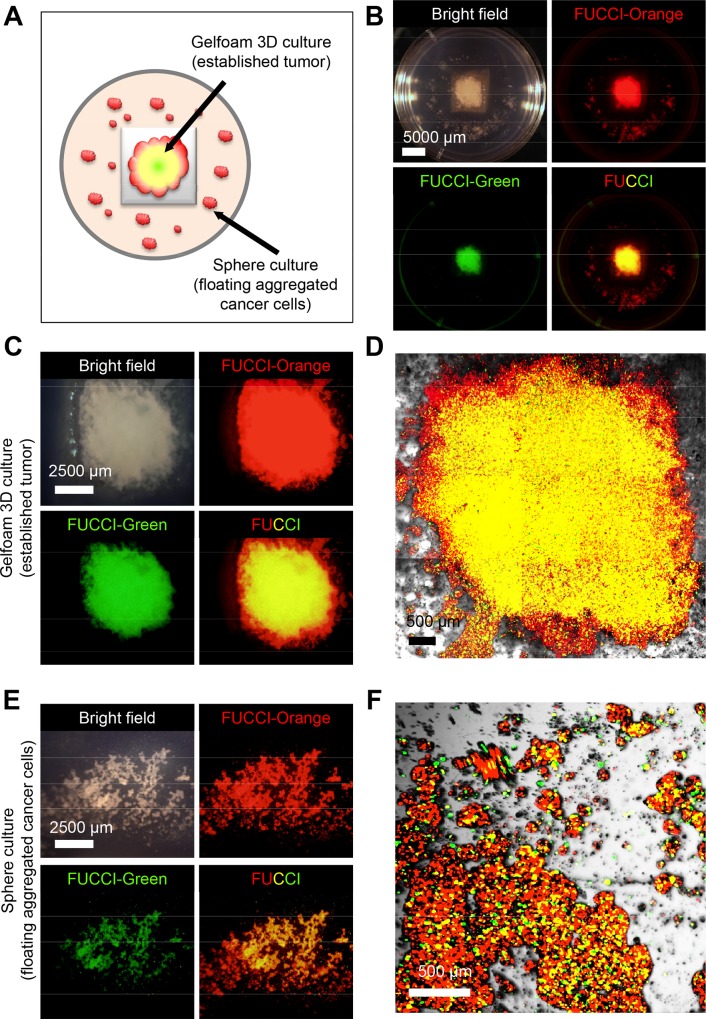
Cell cycle distribution of MKN-45 cells forming tumors on Gelfoam^®^. Sterile Gelfoam^®^ sponges (Pharmacia & Upjohn, Kalamazoo, MI), prepared from porcine skin, were cut into 1 cm cubes. The Gelfoam^®^ cubes were incubated at 37℃ in order that Gelfoam^®^ absorbed the RPMI 1640 medium. The absorbed Gelfoam^®^ was placed on agorose in 35 mm dishes. FUCCI-expressing MKN-45 cells were seeded on the absorbed Gelfoam^®^ for 3D culture. MKN-45 cells formed tumor-like structures on Gelfoam^®^ and spheres on the agarose. **A.** Schema of Gelfoam^®^ cultures on agarose. **B.** Macroscopic appearance of MRN-45 cancer cells forming tumor-like structures on Gelfoam^®^. Images were acquired with the OV100 variable magnification fluorescence imager (Olympus, Japan). **C.** Macroscopic appearance of MKN-45 tumor-like structures growing on Gelfoam^®^. Images were acquired with the OV100. **D.** Representative images of the MKN-45 tumor-like structure on Gelfoam^®^ using the FV1000 confocal laser scanning microscope (Olympus). **E.** Macroscopic images of MKN-45 spheres growing in agar. **F.** Microscopic FUCCI image of MKN-45 spheres.

**Fig 3:**
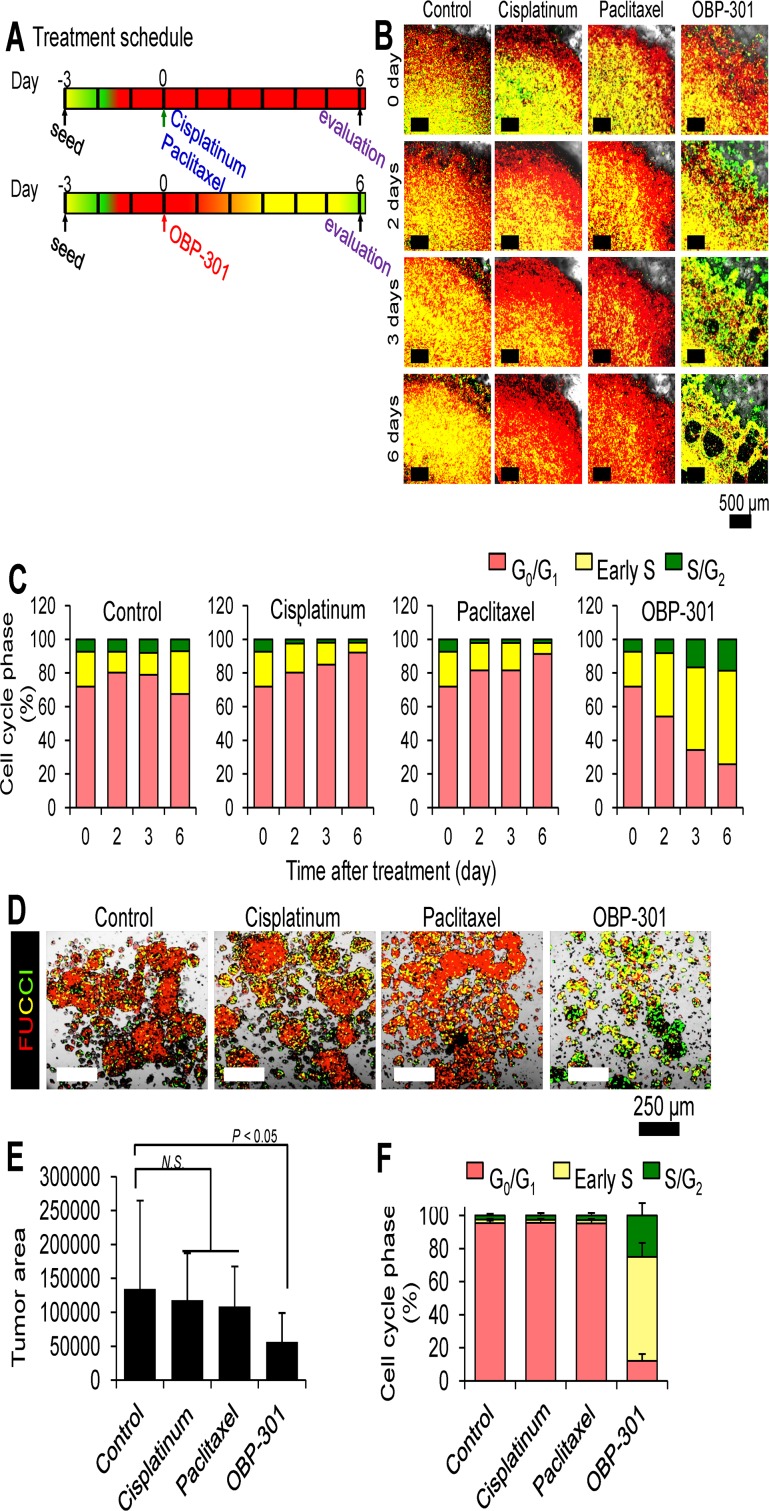
Time-course imaging of decoy of quiescent MKN-45 cancer cells in tumor-like structures on Gelfoam^®^ by OBP-301 and their subsequent killing. **A.** Experimental setup for treatment of MKN-45 tumor-like structures growing in Gelfoam^®^ with OBP-301, CDDP, or paclitaxel. **B.** Time-course images of FUCCI-expressing MKN-45 cancer cells forming tumor-like structures on Gelfoam^®^ treated with OBP-301, CDDP, or paclitaxel. The cells in G_0_/G_1_, S, or G_2_/M phases appear red, yellow, or green fluorescent, respectively. **C.** Histogram shows the cell cycle phase of FUCCI-expressing MKN-45 cancer cells forming tumor-like structures on Gelfoam^®^ treated with OBP-301, CDDP, or paclitaxel. **D.** Representative images of control, OBP-301-, CDDP-, or paclitaxel-treated tumors. **E.** Bar graphs show the size of viable tumor after each treatment. **F.** Histogram shows cell-cycle phase of MKM-45 cells in treated and untreated spheres. Scale bars, 100 μm. Data are shown as means ± SD (n = 5). *P < 0.01. The percentage of cells in G_0_/ G_1_, S, and G_2_/M phases are shown.

FUCCI-expressing MKN-45 cells on Gelfoam^®^ grew into tumor-like structures with the majority of the cells in G_0_/G_1_ (Fig 2) when the tumor-like structure matured. The mature tumors or spheres on Gelfoam^®^ have the vast majority of their cells in G_0_/G_1_ (Figs 1E and 3E and 3F) and thereby look red or yellow. The images are of the whole tumor or sphere, not cross sections. The yellow fluorescence is derived from the mixture of red (G_0_/G_1_) and green (cycling) cells on the surface of the sphere or tumor on Gelfoam^®^.

### OBP-301 Decoyed Quiescent Cancer Cells in Tumors Growing on Gelfoam^®^ to Cycle and Reduced Their Viability

Gelfoam^®^ histocultures of FUCCI-expressing MKN-45 cancer cells were treated with OBP-301, CDDP, or paclitaxel (Fig 3A). Only cycling MKN-45-FUCCI cells were sensitive to CDDP and paclitaxel; quiescent G_0_/G_1_ MKN-45 cells were resistant to these drugs (Fig 3B). In contrast, OBP-301 decoyed the MKN-45 cells in the tumor-like structures on Gelfoam^®^ to cycle from G_0_/G_1_ phase to S/G_2_ phase and reduced their viability (Fig 3B and 3C). CDDP- or paclitaxel-treated MKN-45 tumor-like structure remained quiescent and did not change in size (Fig 3D and 3E). In contrast, OBP-301 reduced the size of the MKN-45 tumor-like structures on Gelfoam^®^ (Fig 3D–3F).

### OBP-301 Modulates Cell-Cycle Related Gene Expression

**Fig 4:**
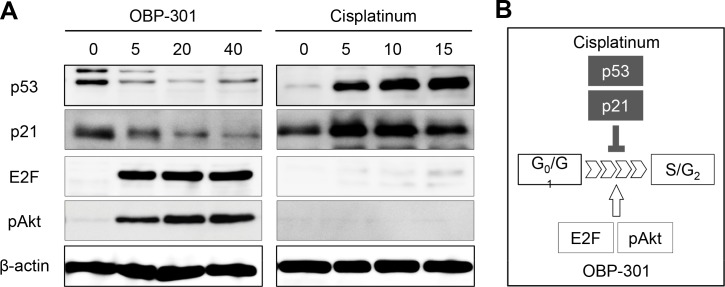
**A.** Western blots show the expressions of p53, p21, E2F, and pAkt after OBP-301 or CDDP treatment. **B.** The scheme of cell cycle change.

We examined the cell cycle-related proteins using Western blotting. CDDP increased the expression of p53 and p21 indicating cell-cycle arrest. In contrast, OBP-301 decreased the expression of p53 and p21 (Fig 4). Furthermore, OBP-301 increased the expression of E2F and pAkt indicating in cell-cycle decoy (Fig 4).

The cell cycle decoy effect of OBP-301 is large scale, whereby the cells in untreated mature spheres or Gelfoam^®^ tumor are almost all in G_0_/G_1_ and almost all of them cycle after OBP-301 infection. The sphere or tumor area is reduced by approximately one half after OBP-301 treatment which is not as great an effect as the extent of decoy. Future experiments will investigate the kinetics of cancer-cell killing after OBP-301 treatment.

## Conclusions

SCCP growing as spheres or tumor-like structures on Gelfoam^®^ cycle became mostly quiescent as they matured and were thereby resistant to chemotherapy. Telomerase-dependent adenovirus OBP-301 could decoy the quiescent tumor cells in spheres or tumor-like structures in Gelfoam^®^ to begin to cycle whereby they lost viability. These results are further demonstration of the power of cell-decoy chemotherapy.

## Abbreviations

FUCCI: fluorescence, ubiquitination-based cell cycle indicator
